# Biomechanical Assessment of a Novel Self-Ligating Orthodontic Bracket Using Finite Element Analysis: The LUMACA System

**DOI:** 10.3390/dj14100664

**Published:** 2026-10-12

**Authors:** Mauro Lorusso, Fariba Esperouz, Michele Tepedino, Ivan Giorgio, Vittorio Mottola, Alessandro Luchetta, Mario Mastrovincenzo, Lucio Lo Russo, Domenico Ciavarella

**Affiliations:** 1 Department of Clinical and Experimental Medicine, Dental School of Foggia, University of Foggia, Via Rovelli, 50, 71122 Foggia, Italy; 2 Department of Biotechnological and Applied Clinical Sciences, Dental School of L’Aquila, University of L’Aquila, 67100 L’Aquila, Italy; 3 Department of Civil, Construction-Architectural and Environmental Engineering (DICEAA), University of L’Aquila, 1, P.zza Ernesto Pontieri, Monteluco di Roio, 67100 L’Aquila, Italy; 4 International Research Center for the Mathematics and Mechanics of Complex Systems (M & MoCS), University of L’Aquila, 1, P.zza Ernesto Pontieri, Monteluco di Roio, 67100 L’Aquila, Italy; 5 Private Practice, 71029 Troia, Italy; 6 Private Practice, 60122 Ancona, Italy; 7 Private Practice, 60126 Ancona, Italy

**Keywords:** finite element analysis, orthodontic biomechanics, LUMACA system, centre of rotation, periodontal ligament

## Abstract

**Objectives:** This study aimed to comparatively assess, using a two-dimensional static finite element analysis (FEA), the initial biomechanical response associated with two LUMACA bracket configurations and a conventional bracket configuration under identical loading and modelling conditions. **Methods:** A two-dimensional finite element model comprising the tooth, cortical and trabecular bone, and periodontal ligament (PDL) was constructed using COMSOL Multiphysics. Three configurations were simulated under a palato-vestibular load: (1) the LUMACA bracket in the standard position, (2) the LUMACA bracket in a gingival (apical) position, and (3) a conventional bracket in the standard position. Each configuration was analysed at five force levels ranging from 0.25 to 2.00 N, with 1.47 N used as the reference load. Von Mises stress, longitudinal and transverse strain distributions, and the displacements of coronal and apical probe points were analysed to characterise the mechanical response and tooth-movement pattern. **Results:** In all three configurations, the crown and apex moved in the same direction and the centre of rotation lay beyond the root apex; therefore, all configurations produced controlled tipping. The apex-to-crown ratio of the displacement components along the applied load, which equals 1 for pure bodily translation and 0 for rotation about the apex, was 0.771 for the gingivally bonded LUMACA bracket, 0.469 for the LUMACA bracket in the standard position, and 0.227 for the conventional bracket. The corresponding centres of rotation were located 37.0, 9.7, and 3.2 mm beyond the root apex, respectively. Under a load of 1.47 N, the differential displacement between the crown and apex was 0.050, 0.211, and 0.516 micrometres, respectively; thus, the conventional bracket produced approximately ten times the differential displacement observed with the gingivally bonded LUMACA bracket. The response was linear across the entire force range examined, indicating that the ranking of the three configurations was independent of load magnitude. **Conclusions:** Bracket slot position substantially influenced the biomechanical response of the tooth–PDL complex. The apically bonded LUMACA bracket displaced the centre of rotation farther beyond the root apex and produced a more uniform PDL deformation pattern than the conventional design. The calculated stress distributions and displacements should be interpreted as comparative metrics between bracket configurations and not as direct predictors of clinical outcomes.

## 1. Introduction

The precise control of tooth movement in relation to the centre of resistance is still a critical issue in modern Orthodontics, and excessive or misdirected forces can compromise treatment outcomes. Furthermore, while new bracket systems continue to be introduced, comparative evidence regarding their biomechanical effectiveness is limited. This highlights the need for an interdisciplinary approach that integrates biomechanics, materials science, and clinical practice, particularly considering that orthodontic treatment may be influenced by several variables, including some that are often overlooked, such as individual differences in occlusal force or unplanned changes in the curve of Spee during treatment [1,2]. Increasing patient expectations for efficient, aesthetic, and biologically sound treatment further emphasise the relevance of this field [3,4,5]. The pursuit of improved biomechanical control has led to the development of numerous orthodontic techniques. In the mid-1990s, the American orthodontist Dwight Damon introduced a self-ligating bracket system [6], combined with copper–nickel–titanium archwires designed to reduce slot friction and facilitate tooth movement. This approach was intended to facilitate the early engagement and alignment of teeth positioned substantially away from the occlusal plane [7], challenging some traditional concepts of the straight-wire technique [8] and the geometric principles described by Burstone [9]. Over time, clinicians and industry partners have developed a wide range of bracket systems based on different biomechanical philosophies [10]. This diversity provides clinicians with multiple treatment options but also underscores the need for comparative biomechanical evidence. In parallel, clear aligners have become an increasingly used alternative to fixed appliances, largely because of patient demand for improved aesthetics and comfort, although fixed appliances and aligners may differ in their biomechanical and clinical effects [11,12,13].

In addition to manufacturing tolerances, bracket placement accuracy is an important determinant of prescription expression because vertical and angular positioning errors can alter torque expression, force-application geometry, and the resulting tooth movement [14]. Torque expression is also influenced by dimensional variability within the bracket–slot and archwire system. Tepedino et al. [15] reported deviations between nominal and actual slot–archwire dimensions, which increase torsional play and may reduce the precision of torque expression during orthodontic treatment.

In response to these biomechanical challenges, Ciavarella, Mastrovincenzo, and Luchetta developed the LUMACA bracket, a self-ligating system designed to modify the line of action of orthodontic forces. Its geometry is intended for clinical situations in which conventional bracket positioning may generate undesirable rotational moments, such as proclined maxillary incisors. Whereas conventional straight-wire or MBT brackets position the slot close to the tooth surface, the LUMACA system relocates the slot several millimetres away from the crown. This geometric modification alters the height of force application and the associated lever arm. By modifying this lever arm, the LUMACA design is intended to reduce the rotational component of tooth movement and alter the distribution of mechanical loading within the periodontal ligament. Even relatively small changes in bracket geometry and slot position may substantially affect stress and strain distributions within the periodontal ligament and alveolar bone. These biomechanical effects can be investigated systematically using finite element analysis (FEA).

The integration of FEA with in silico and patient-specific models for investigating the influence of mechanical forces on tissue adaptation has been described within the framework of Theramechanics. This approach provides a potential means of linking mechanical loading conditions with biological responses in the periodontal ligament (PDL) and alveolar bone. Such models may support the identification of loading strategies associated with specific tissue responses and may contribute to the development of more targeted orthodontic approaches [16,17].

The aim of the present study was to investigate, using finite element analysis, the biomechanical effects of two LUMACA bracket configurations compared with a conventional bracket.

## 2. Materials and Methods

**Figure 1:**
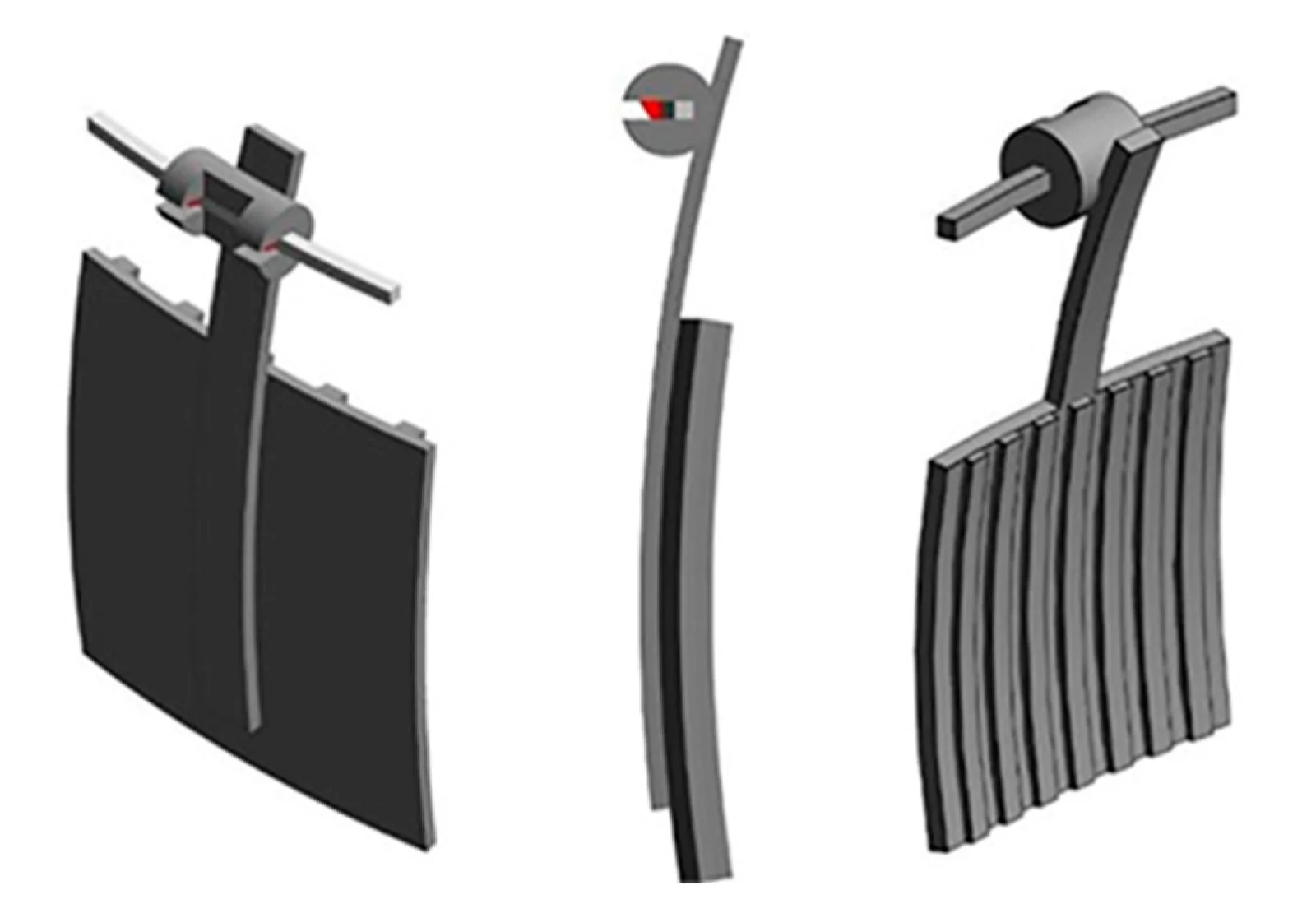
Design of the LUMACA bracket.

The finite element analysis explored the biomechanical implications of the positional alignment of bracket slots on dental displacement and the resultant stress–strain distribution within the periodontal ligament (PDL) when subjected to a palato-vestibular force of about 1.5 N. The investigations were performed by means of COMSOL Multiphysics (version 6.4.0.429, COMSOL AB, Stockholm, Sweden) simulations of three configurations: (1) the LUMACA bracket bonded in the standard position, (2) the LUMACA bracket bonded in a more gingival position, and (3) a conventional bracket bonded in the standard position. Figure 1 shows the structural design and distinctive characteristics of the LUMACA bracket.

## 3. LUMACA System

The LUMACA bracket is a novel self-ligating system conceived and developed by Ciavarella, Mastrovincenzo, and Luchetta. The bracket base measures 4.0 mm in width and 2.5 mm in height, with an in-out dimension of 1.0 mm. An arm extending 2.5–3.5 mm from the base supports the slot and determines its spatial position relative to the tooth surface.

The finite element model used a two-dimensional plane-strain approximation of a sagittal cross-section comprising the tooth, maxillary bone, and periodontal ligament (PDL). The model assumes that the applied force generates negligible stress and deformation in the direction orthogonal to the plane of the section and that local motion can therefore be approximated as planar. This represents a substantial modelling assumption and introduces inherent limitations. Nevertheless, the resulting computational simplification permits a controlled preliminary comparison of the different configurations investigated.

The analysed geometry represented a maxillary central incisor sectioned along its long axis in the sagittal plane, together with the surrounding periodontal ligament and the cortical and trabecular bone of the alveolar process; the palato-vestibular load therefore acted within the plane of the section. The geometry was not patient-specific and was not derived from CT or CBCT imaging. Instead, an idealised two-dimensional profile was constructed to reproduce average anatomical proportions of the tooth, alveolar socket, and surrounding bone reported in the literature. The periodontal ligament was represented as an approximately uniform 0.2-mm layer surrounding the root, although its thickness varies along the root in vivo. This idealisation was adopted deliberately because the objective was to compare three bracket configurations under otherwise identical conditions rather than to reproduce patient-specific anatomy.

The model comprised cortical bone, trabecular bone, the tooth, and the periodontal ligament. The tooth was modelled as a homogeneous structure because its substantially greater stiffness relative to the PDL meant that its internal composition was not expected to materially affect the comparative analysis. The PDL was represented as the connective interface between the tooth root and the alveolar socket. Each component was modelled as a homogeneous continuum using the elastic properties listed in Table 1. Tooth and bone were modelled as linear isotropic materials, whereas the PDL was represented as an orthotropic elastic material (Table 2) to account for its fibre-oriented substructure. The principal material directions were aligned transversely across the ligament (direction 1) and longitudinally along it (direction 2), with the third axis completing the orthogonal reference frame. Material properties were fixed and single-valued for all components. Geometric nonlinearity, accounting for large displacements and rotations, was enabled only for the PDL, which was the component expected to undergo appreciable deformation. No material nonlinearity, viscoelasticity, or load-history dependence was introduced. As shown in the Results, the geometrically nonlinear contribution was negligible at the load levels examined: the computed response remained proportional to the applied force within 0.006% across an eightfold load range. The model therefore behaved effectively linearly within the investigated regime.

**Figure 2:**
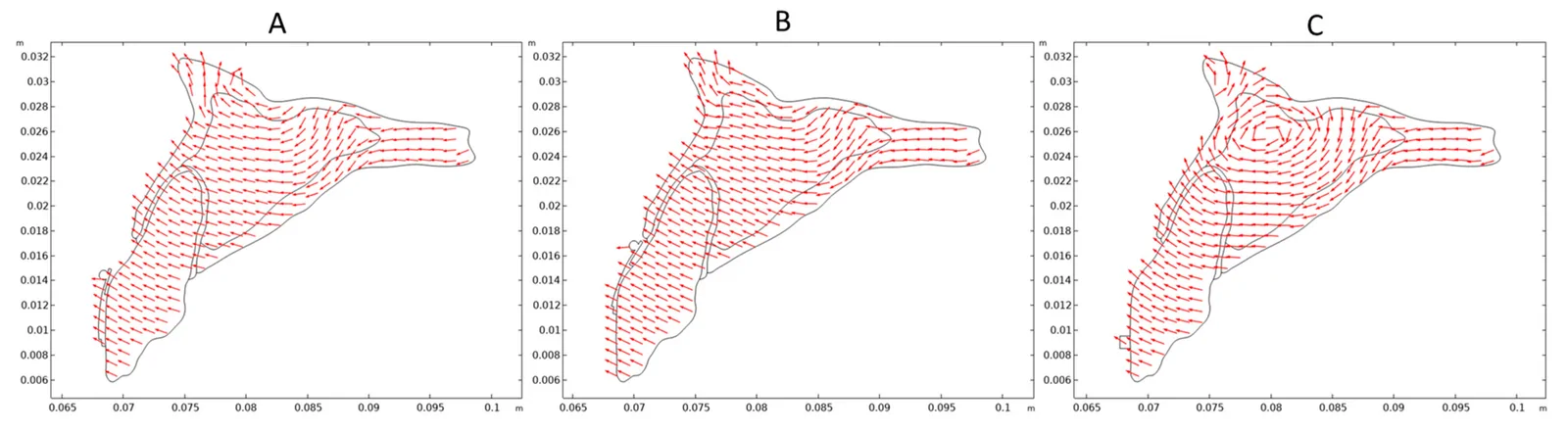
Vectors indicating the direction of tooth displacement under force application with a Lumaca bracket in the standard position (**A**) and in a gingival position (**B**) and with a conventional bracket in the standard position (**C**).

**Figure 3:**
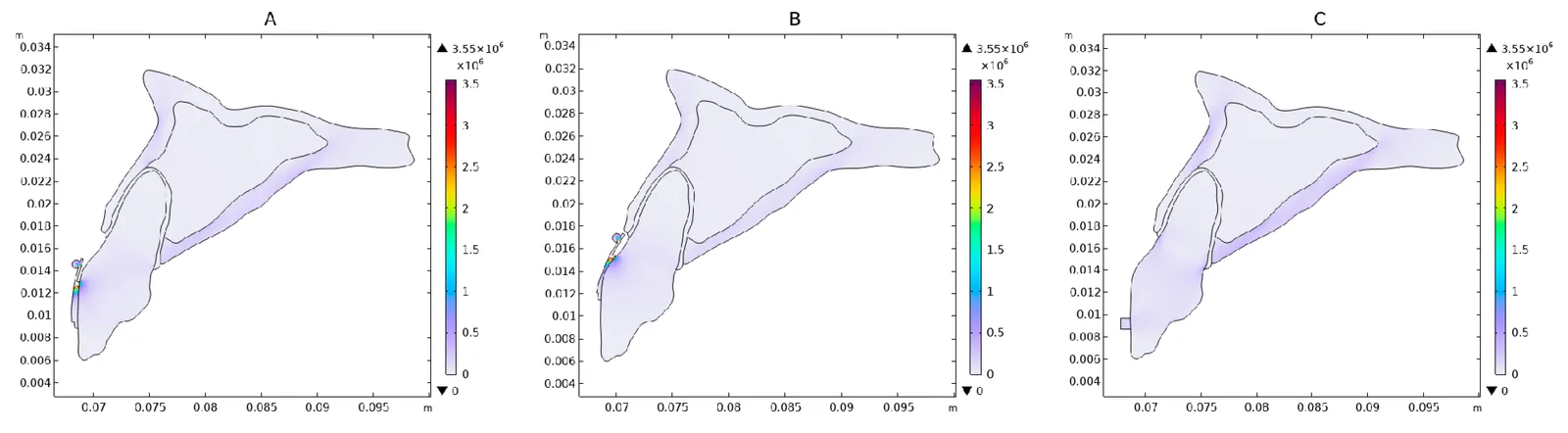
Von Mises stress in the whole model under force application with a Lumaca bracket in the standard position (**A**) and in gingival position (**B**) and with a conventional bracket in the standard position (**C**). Values in Pa; the three panels share the same colour scale to allow direct comparison.

**Figure 4:**
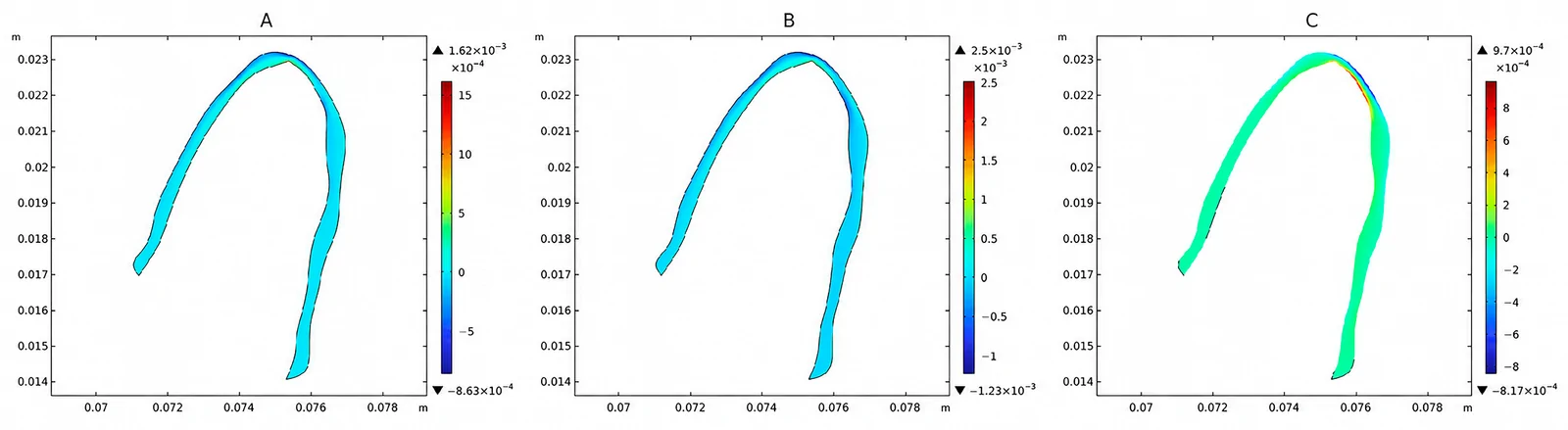
Longitudinal deformation of the periodontal ligament under force application with a Lumaca bracket in the standard position (**A**) and in a gingival position (**B**) and with a conventional bracket in the standard position (**C**).

**Figure 5:**
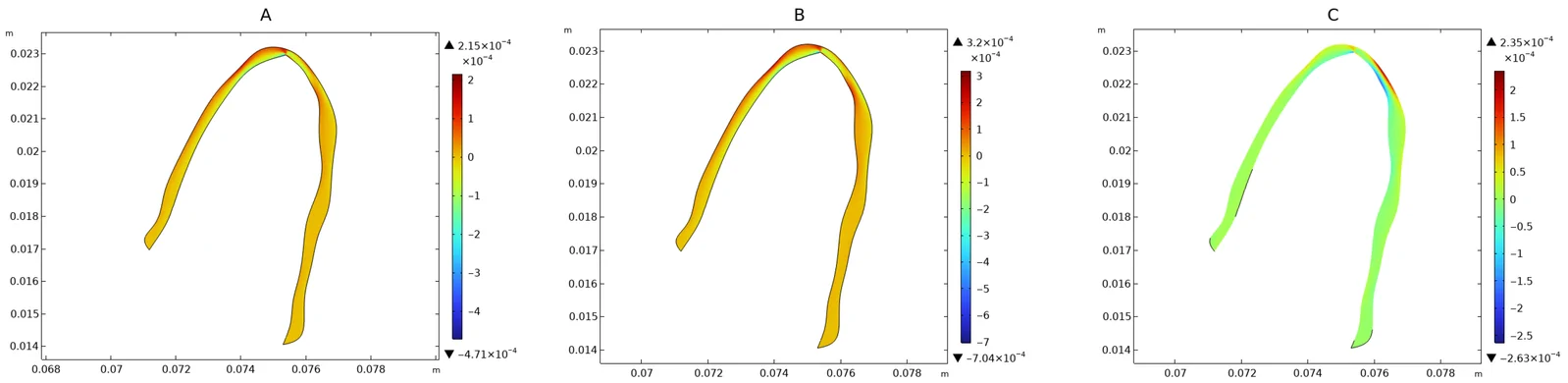
Transversal deformation of the periodontal ligament under force application with a Lumaca bracket in the standard position (**A**) and in a gingival position (**B**) and with a conventional bracket in the standard position (**C**).

**Figure 6:**
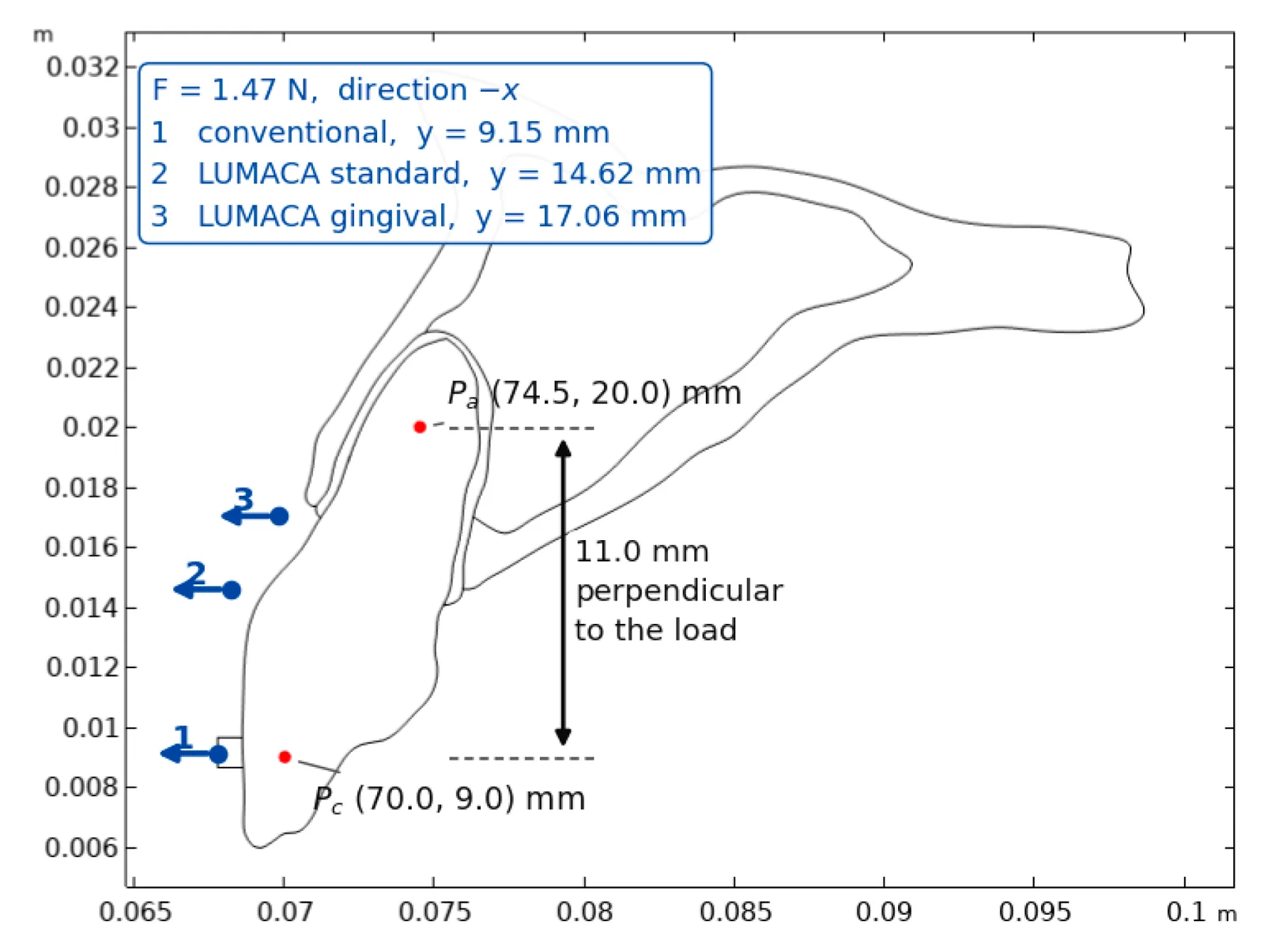
Model set-up. The two red markers are the probe points used to assess tooth displacement: the coronal probe Pc at (70.0, 9.0) mm and the apical probe Pa at (74.5, 20.0) mm, whose separation in the direction perpendicular to the applied load is 11.0 mm. The blue arrows indicate the point of application and the direction of the 1.47 N palato-vestibular load in the three bracket configurations: (1) conventional bracket, line of action at coordinate y = 9.15 mm; (2) LUMACA bracket in the standard position, y = 14.62 mm; (3) LUMACA bracket in the gingival position, y = 17.06 mm. All coordinates refer to the model reference frame.

**Figure 7:**
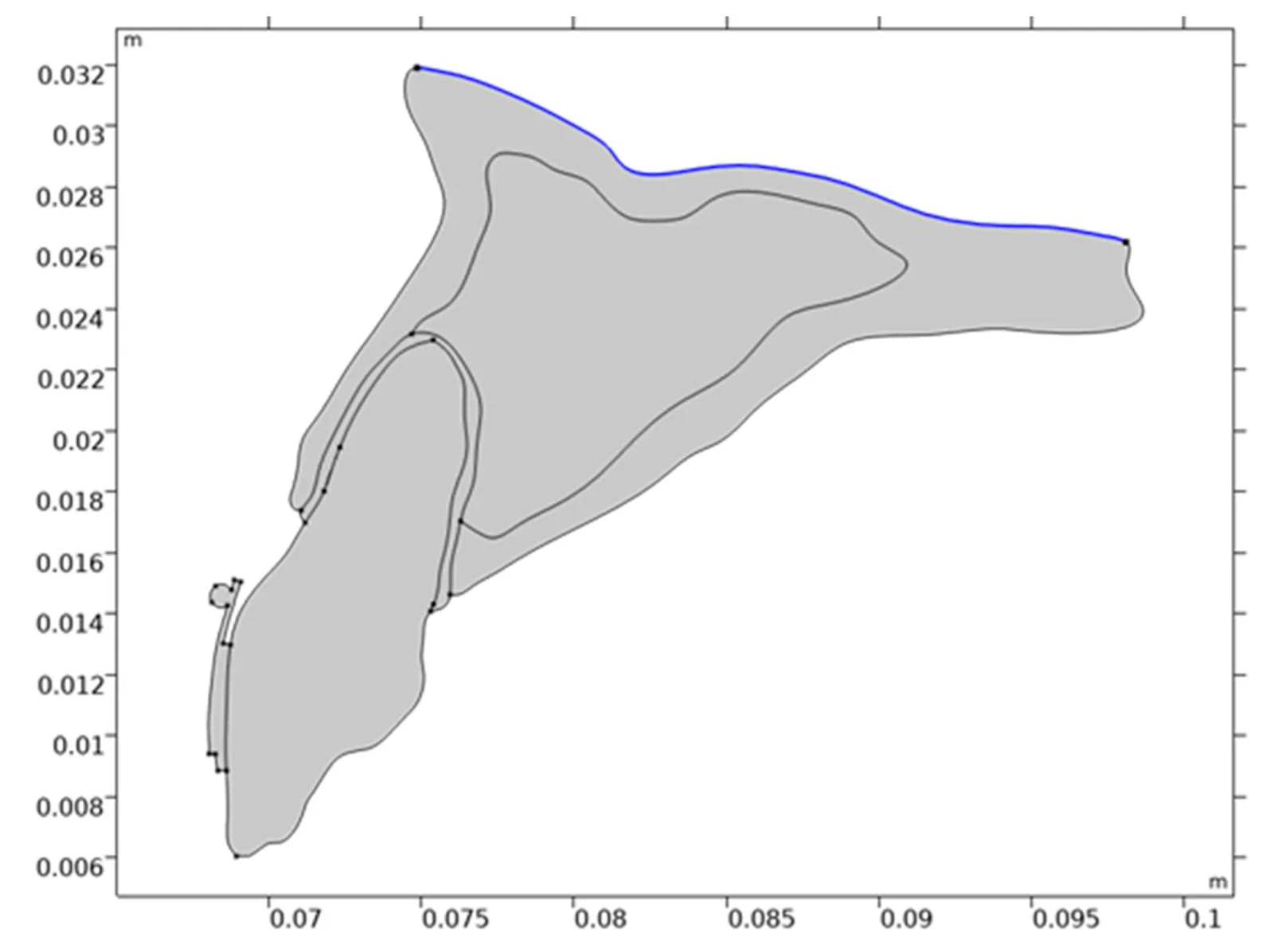
Geometry of the model with the constrained boundary highlighted in blue; a zero-displacement constraint is imposed on the outer superior boundary of the maxillary bone. All other boundaries are free.

In the two-dimensional solid-mechanics formulation, the nodes have only translational degrees of freedom; therefore, prescribing zero displacement along this edge is equivalent to a fully fixed constraint. The constrained boundary is remote from the region of interest relative to the thickness of the PDL, thereby limiting its influence on ligament response. All remaining boundaries were left free. A force of 1.47 N (150 g-force) was applied at the bracket slot in the palato-vestibular direction within the plane of the section. This load represents the force transmitted by an engaged archwire. No couple was applied at the slot, and the implications of this assumption are discussed in the Limitations. Figure 2 illustrates the displacement vector fields resulting from force application. The analysis included von Mises stress (Figure 3A–C), longitudinal and transverse strain within the PDL (Figure 4A–C and Figure 5A–C, respectively), and displacement at two probe points located on the crown and root apex (Figure 6). A zero-displacement constraint was imposed on the outer superior boundary of the maxillary bone, highlighted in blue in Figure 7.

The discretisation was verified via a convergence analysis. Four meshes were generated for the LUMACA bracket in the standard position under a load of 1.47 N, with maximum element sizes of 0.1, 0.0667, 0.05 and 0.033 mm, corresponding to approximately three, six, eight and twelve second-order (quadratic) Lagrangian elements across the thickness of the periodontal ligament and to between 3.1 × 10^5^ and 2.8 × 10^6^ degrees of freedom (Table 3). Convergence was monitored on three quantities: the displacement of the coronal probe point *u_c_*, the apex-to-crown displacement ratio *r*, and the resultant of the traction integrated over the tooth–ligament interface. The last of these is a particularly severe test because its exact value is known a priori: global equilibrium of the tooth requires the integrated traction to equal the applied load so that its departure from 1.47 N is a direct measure of the discretisation error and admits no adjustable interpretation. The results are collected in Table 4. The kinematic quantities are already converged on the coarsest mesh: over the whole refinement sequence, in which the number of degrees of freedom increases by a factor of nearly nine, the crown displacement varies by 0.011% and the apex-to-crown displacement ratio by 0.002%. The integrated traction converges monotonically towards the applied load; the error falls from 6.57% to 0.84%, with an observed rate of approximately 1.5, which is below the nominal order of the elements employed, as is to be expected in the presence of the higher-contrast stiffness at the alveolar crest (refer to [18,19,20] for additional information on how to address this issue). The descriptors on which the conclusions of this study rest are therefore mesh independent to four significant figures, whereas the quantities derived from the local stress field converge appreciably more slowly; this is a further reason for basing the comparison among the three configurations on the kinematic descriptors, as is done in the following sections. The results reported in this work were obtained with the second mesh of the sequence, which provides five to six elements across the ligament, and are unchanged, to the digits quoted, on the two finer meshes.

To assess the influence of the magnitude of the applied load, each of the three bracket configurations was additionally analysed at five force levels, namely 0.25, 0.50, 1.00, 1.47 and 2.00 N, which span the range of forces clinically employed for the movement of a single incisor and extend above it. Geometry, mesh, material parameters and boundary conditions were left unchanged, so the magnitude of the applied force was the only variable. The periodontal ligament was discretised with five to six elements across its thickness, using second-order (quadratic) Lagrangian elements, and its thickness was kept within the physiological range reported in the literature. Since the applied load is directed along the palato-vestibular direction, all the descriptors of the movement are defined in terms of the displacement component along that direction and of distances measured perpendicular to it. Three descriptors were derived from the displacement of the coronal and apical probe points: the apex-to-crown ratio of the displacement components along the load, *r* = *u_a_*/*u_c_*; the position of the centre of rotation, obtained as the point at which the displacement component along the load vanishes; and the rotation of the tooth per unit of applied force. Being ratios of quantities that scale linearly with the load, these descriptors are independent of the force magnitude, as the results below confirm.

## 4. Results

Figure 2 illustrates the finite element analysis of a 1.47 N palato-vestibular load. In Figure 2A, the LUMACA bracket slot is positioned apically relative to the geometric centre of the bracket body. The displacement field exhibits nearly parallel vectors, with minimal rotation of both the crown and the root. Under the assumptions of the present model, this configuration showed a smaller rotational component and a displacement pattern closer to bodily translation than the conventional configuration. In Figure 2B, the LUMACA bracket slot is positioned more apically than in Figure 2A, bringing the line of action of the archwire force closer to the centre of resistance. This configuration showed a further reduction in the rotational component, resulting in an initial displacement pattern closer to bodily translation. The displacement field showed more nearly parallel vectors and the smallest rotational component among the three simulated configurations, corresponding to an initial displacement pattern closer to bodily translation.

In Figure 2C the slot is located centrally within the bracket body, as in conventional bracket systems. Here the line of action of the archwire force lies further from the centre of resistance, thereby increasing the moment arm and inducing a larger rotational component. The displacement field displays a clear rotational pattern about a centre of rotation situated close to the root apex, the crown moving vestibularly and the apex following in the same direction but through a much smaller displacement. This configuration showed a greater rotational component than the two LUMACA configurations, as reflected by the smaller apex-to-crown displacement ratio and the centre of rotation located closer to the root apex. It should be noted that the present analysis does not compute the moment-to-force ratio, since the load applied is a single force and the position of the centre of resistance is not determined here; the quantity measured is the position of the centre of rotation, from which the type of movement is characterised.

Figure 3 shows the Von Mises stress over the whole model, with a common colour scale for the three configurations, so that the path followed by the applied load can be appreciated. In every case, the stress is concentrated at the interface between the bracket and the crown, where the load is introduced, and decays rapidly within the tooth, while the cortical and trabecular bone remain lightly stressed throughout, the values there being well below one megapascal. The maximum, 3.55 MPa, occurs at the bracket-to-crown interface of the two LUMACA configurations and reflects the longer lever arm of that design, which relocates the slot several millimetres from the crown surface; it is a local value at a geometric corner and remains more than two orders of magnitude below the yield strength of titanium, although the adequacy of the adhesive bond under the increased moment is a question for experimental validation rather than for the present model. The periodontal ligament, being three to four orders of magnitude more compliant than the surrounding tissues, carries stresses far lower than the scale of this figure and is therefore not resolved in it; its mechanical response is examined through the strain fields of Figure 4 and Figure 5 and, quantitatively, through the kinematic descriptors reported below. Absolute stress magnitudes within the ligament are not quoted, since its extreme values are located where the layer is thinnest and where the elastic solution is less accurate because of higher-contrast stiffness compared to the surroundings, so they depend on discretisation.

Figure 4 shows the distribution of the longitudinal strain within the periodontal ligament. In Figure 4A the tensile regions are located coronally on the vestibular side and the compressive regions apically on the palatal side, with a smooth and nearly symmetrical distribution. In Figure 4B, with the slot placed more gingivally, the altered line of force application was associated with a more homogeneous strain distribution along the root, with a reduced gradient between the apical and cervical regions. In Figure 4C, the larger moment produces a markedly asymmetric field, with pronounced tension in the coronal vestibular area and compression in the apical palatal region, a pattern indicative of a centre of rotation close to the root apex. Strain is a dimensionless quantity; under a load of 1.47 N its extreme values are of the order of 10^−3^ and are located where the ligament is thinnest. These extremes do not order in the same way as the movement pattern, being largest for the gingivally bonded configuration and smallest for the conventional one. This is the expected consequence of global equilibrium: the same force is transmitted across the same ligament in the three cases, so a movement closer to bodily translation engages a greater portion of the ligament and cannot reduce the total load that the ligament carries. For this reason, they are not used as a basis for comparison; the uniformity of the loading is instead quantified below through the position of the centre of rotation.

Figure 5 illustrates the transverse strain within the periodontal ligament for the three bracket configurations, which is the lateral deformation of the ligament perpendicular to the tooth long axis. In Figure 5A the field is symmetrical and well balanced, with the higher tensile values being observed along the coronal vestibular side and mild compression observed in the apical palatal region. When the slot is placed more gingivally (Figure 5B), the altered force-application geometry is associated with a smaller rotational component and a more uniform transverse strain distribution along the root, with more balanced tensile and compressive regions and smaller regional gradients. In contrast, when the slot is centred within the conventional bracket body, Figure 5C, the field is strongly asymmetric: the coronal vestibular area shows evident tensile strain while the apical palatal side undergoes stronger compression, and the gradient along the root is the largest of the three configurations.

The comparison of the three transverse strain fields reproduced the relative ordering observed from the displacement-based descriptors: within the present model, the gingivally bonded LUMACA configuration showed the most uniform PDL deformation pattern, the standard LUMACA configuration showed intermediate characteristics, and the conventional configuration exhibited the greatest deformation gradient along the root. It should be noted that in none of the three configurations do the crown and the apex move in opposite directions, so none of them corresponds to uncontrolled tipping in the classical sense; the difference between them lies in how far the centre of rotation is displaced beyond the root apex, as quantified in the following section.

To evaluate how bracket slot position influences the pattern of tooth movement, two probe points were selected, one located on the coronal region of the tooth close to the bracket area and one on the apical region near the root apex (Figure 6), their separation in the direction perpendicular to the applied load being 11.0 mm. For each bracket configuration, the displacement component along the direction of the load was extracted at both points. In all three configurations the two components have the same sign, the crown moving more than the apex, so the centre of rotation lies beyond the root apex and the movement is of the controlled tipping type, approaching bodily translation as the slot is moved gingivally. Under a load of 1.47 N the crown and apex displacements are 0.219 and 0.169 micrometres for the LUMACA bracket bonded in the gingival position, 0.398 and 0.187 micrometres for the LUMACA bracket in the standard position, and 0.668 and 0.152 micrometres for the conventional bracket, corresponding to apex-to-crown ratios of 0.771, 0.469 and 0.227 respectively. The differential displacement between the two points, which measures the departure from bodily movement, is 0.050, 0.211 and 0.516 micrometres: the conventional bracket therefore produces about ten times the differential displacement of the apically bonded LUMACA bracket, and the LUMACA bracket in the standard position produces about four times more displacement.

### Influence of the Magnitude of the Applied Force

Table 3 reports the displacement of the coronal and apical probe points for the three bracket configurations at the five force levels considered. In every configuration, the displacements are proportional to the applied load: the crown displacement per unit force varies by less than 0.006% over the whole range, and the apex-to-crown ratio by less than 0.004%. The response of the model is therefore linear over a force range spanning a factor of eight, and the type of movement produced by each configuration does not depend on the magnitude of the force applied.

The centre of rotation, obtained as the point at which the displacement component along the load vanishes, lies beyond the root apex in all three configurations, so the ratio *r* is bounded between zero and one and measures directly how closely the movement approaches bodily translation, with *r* = 1 corresponding to pure translation and *r* = 0 corresponding to rotation about the apex. The values obtained are 0.771 for the LUMACA bracket bonded in the gingival position, 0.469 for the LUMACA bracket in the standard position and 0.227 for the conventional bracket. The corresponding centres of rotation lie 37.0, 9.7 and 3.2 mm beyond the root apex, measured perpendicular to the direction of the applied load, and the rotation produced per unit of applied force is 3.10 × 10^−6^, 1.31 × 10^−5^ and 3.19 × 10^−5^ rad/N, respectively. For the same applied force, the conventional bracket thus rotates the tooth about ten times more than the apically bonded LUMACA bracket and about two and a half times more than the LUMACA bracket in the standard position. All these quantities are ratios of quantities proportional to the load and are therefore independent of its magnitude, so the ranking of the three configurations is preserved at every level examined.

Because the deformation of the periodontal ligament is proportional to the distance from the centre of rotation, the position of the latter also determines how uniformly the ligament is loaded along the root. The root extends 7.4 mm along its axis, which is 6.85 mm in the direction perpendicular to the applied load, and the ratio between the smallest and the largest ligament strain along the root, U, is accordingly 0.844 for the LUMACA bracket bonded in the gingival position, 0.587 for the LUMACA bracket in the standard position and 0.321 for the conventional bracket, where U = 1 corresponds to a perfectly uniform loading of the ligament that is to bodily translation and U = 0 to corresponds rotation about the apex. Equivalently, the ratio between the peak and the mean traction transmitted to the alveolar wall is 1.09, 1.26 and 1.51, respectively. The apically bonded configuration therefore engages the ligament almost uniformly along the whole root, whereas the conventional bracket concentrates the load in the cervical third. This result follows directly from the measured kinematics and does not require the evaluation of local stress peaks, which are less accurate.

## 5. Discussion

Finite element method (FEM) modelling is widely used to investigate orthodontic biomechanics because it enables detailed evaluation of stress, strain, and displacement within the tooth–PDL–bone complex under controlled loading conditions.

Orthodontic tooth movement results from the combined effects of translation and rotation. Depending on the applied force system, the initial displacement pattern may correspond to uncontrolled tipping, controlled tipping, translation, or root movement. From a mechanical perspective, the moment-to-force (M/F) ratio is a key determinant of the resulting centre of rotation and displacement pattern [21]. The centre of resistance is the conceptual point through which a pure force would produce translation without rotation [22], and its position depends on tooth geometry and the mechanical properties of the root, PDL, and surrounding bone. Orthodontic loading also generates mechanical signals within the PDL and alveolar bone that initiate cellular and molecular pathways involved in tissue remodelling and tooth movement [23,24,25].

Appropriate control of orthodontic force magnitude is essential because excessive loading may damage periodontal tissues and the tooth. A central biomechanical challenge in fixed-appliance therapy is the distance between the point of force application and the tooth’s centre of resistance. Because brackets are bonded to the crown, even a more gingival bracket position remains coronal to the centre of resistance, whose location varies with root and supporting-tissue anatomy. When a force acts away from the centre of resistance, it generates a moment equal to the product of the force magnitude and the perpendicular distance from its line of action to the centre of resistance [26].

Experimental data provide reference mechanical properties for dental and periodontal tissues. Cortical bone typically has a Young’s modulus (E) of 17.3 GPa and a Poisson ratio (nu) of 0.28, whereas trabecular bone has an E of approximately 0.64 GPa and a nu of 0.28 [27]. Enamel exhibits elastic moduli of approximately 73–88 GPa depending on prism orientation [28], whereas dentine values range from 11 to 20 GPa and vary towards the dentino-enamel junction [28]. Poisson’s ratio is commonly assigned values of approximately 0.30–0.33 for enamel [27,29], although experimental studies demonstrate material variability and anisotropy, particularly in dentine (0.025 to 0.26) [30,31].

The PDL is particularly challenging to model because of its complex, highly nonlinear, load-dependent mechanical behaviour [32,33]. PDL thickness is also an important determinant of the biomechanical response of the tooth–PDL–bone complex. A thinner ligament tends to produce greater local stress concentrations, whereas a thicker ligament distributes loading over a larger region. Human studies report PDL thicknesses of approximately 0.1–0.24 mm [29,34,35]. Although this range is narrow in absolute terms, finite element studies indicate that it can substantially affect predicted stress distributions. Models using PDL thicknesses closer to 0.1–0.15 mm predict higher peak stresses in the apical root and alveolar socket regions, a mechanical condition that has been discussed as potentially relevant to periodontal loading and root resorption [29,34]. Conversely, thicker PDL representations (0.20–0.24 mm) generally produce lower local stress concentrations and more uniform force distributions [29,35]. Incorporating physiologically realistic PDL thickness values is therefore important for improving the biomechanical fidelity of simulated tooth movement and periodontal loading. Although local stress concentrations have been discussed in previous studies in relation to orthodontically induced root resorption, the present static FEA does not reproduce the biological mechanisms underlying this process. Therefore, the observed differences in stress and strain distribution cannot be interpreted as evidence of either an increased or reduced risk of root resorption.

Given the porous nature of the PDL and its interaction with interstitial fluids, poroelastic formulations may provide a more realistic representation of its mechanical behaviour [36,37]. Incorporating dissipative phenomena [38,39], and fibre architecture through generalised continuum models [40,41,42,43,44] may further improve model fidelity. Within such frameworks, accounting for patient-specific variability and uncertainty may also improve the robustness of biomechanical assessments and treatment-planning models [45,46].

Orthodontic appliances generate stresses and strains within the PDL and alveolar bone. These mechanical stimuli are transmitted through mechanotransduction pathways, activating cellular responses that contribute to tissue remodelling and orthodontic tooth movement [47].

The von Mises stress distribution varied with slot position primarily in the spatial pattern of loading rather than in its overall magnitude. Because the same force was transmitted through the same ligament in all three configurations, global equilibrium constrained the mean traction carried by the PDL; the principal difference therefore lay in how this load was distributed along the root. In the gingival and standard LUMACA configurations, loading extended along a greater portion of the ligament with more moderate gradients, indicating a more even distribution. This behaviour is consistent with previous findings indicating that more apical force application can reduce the moment arm and produce a more homogeneous PDL stress pattern [48]. By contrast, the conventional configuration concentrated loading in the cervical region while a larger portion of the ligament remained comparatively less engaged, consistent with a greater bending moment [49]. Similar localised concentrations have been reported in finite element studies of bracket–slot mechanics and force systems [50,51]. Extreme field values occurred near the alveolar crest and root apex, where the elastic solution is more sensitive to mesh discretisation; these values were therefore not used as the primary basis for comparison. Accordingly, the stress distributions observed in the present study should be interpreted as comparative mechanical patterns between configurations rather than as indicators of periodontal tissue damage, biological safety, or clinical effectiveness.

The strain analysis provided complementary information on PDL deformation. Because the tooth behaves approximately as a rigid body relative to the compliant ligament, local PDL deformation varies with distance from the centre of rotation. The ratio between the minimum and maximum ligament strain along the root (U) was 0.858 for the gingivally bonded LUMACA bracket, 0.603 for the standard LUMACA position, and 0.344 for the conventional bracket, where U = 1 represents perfectly uniform deformation and U = 0 represents rotation about the apex. In both LUMACA configurations, longitudinal and transverse strain showed smoother gradients and more balanced regions of tension and compression, whereas the conventional centred-slot configuration produced a more asymmetric field, with deformation concentrated in the cervical third. Comparable deformation patterns have been described in previous finite element studies of modified slot geometries [52], while non-uniform deformation fields have also been discussed in relation to periodontal loading and alveolar bone remodeling [53]. Importantly, the biomechanical difference identified here concerns the uniformity of deformation along the root rather than a reduction in peak strain. Because the applied force was identical across configurations, the total load transmitted through the PDL was also identical; the configurations differed primarily in how that load was distributed. This difference in deformation distribution should not be interpreted as evidence of a biologically safer loading condition. The present static model cannot determine whether these mechanical differences would result in different cellular responses, periodontal outcomes, rates of orthodontic tooth movement, or treatment-related adverse effects in vivo.

Probe-point displacements confirmed these trends quantitatively. With the LUMACA bracket bonded in the gingival position, crown and apex displacements were relatively similar, with an apex-to-crown ratio of 0.771 and a differential displacement of only 0.050 micrometres under 1.47 N, consistent with a movement pattern approaching bodily translation. Harsha et al. [54] likewise reported that force systems more closely aligned with the centre of resistance reduce coronal–apical displacement differences and rotational components. With the LUMACA bracket in the standard position, differential displacement increased to 0.211 micrometres and the ratio decreased to 0.469, indicating a larger rotational component. The conventional bracket produced the largest differential displacement (0.516 micrometres) and the lowest ratio (0.227), with the centre of rotation located 3.2 mm beyond the root apex. The progressive reduction in the apex-to-crown ratio from the gingival LUMACA configuration to the conventional bracket demonstrates that slot position strongly influences the centre of rotation and the resulting movement pattern. A more apical slot produced a movement pattern closer to bodily translation and a more uniform PDL deformation pattern, whereas the conventional centred slot increased the rotational component and concentrated deformation cervically. These findings describe the relative initial kinematic response predicted by the static model and do not establish that the same magnitude, pattern, or degree of tooth-movement control would occur during clinical orthodontic treatment.

Taken together, the stress, strain, and displacement findings demonstrate consistent relative differences among the three bracket configurations under identical modelling and loading conditions. Within the present 2D static FEA model, the gingivally bonded LUMACA configuration was associated with a centre of rotation located farther beyond the root apex, a more uniform PDL deformation pattern, and smaller crown–apex differential displacement and rotation per unit force than the standard LUMACA and conventional configurations. The standard LUMACA configuration showed intermediate values for these descriptors, whereas the conventional configuration showed the largest rotational component. These relative differences are consistent with the mechanical effects expected from modifying the geometry of force application [48]. However, they describe only the initial static mechanical response predicted by the model and do not demonstrate superior orthodontic treatment performance, greater predictability of bodily tooth movement in vivo, improved periodontal safety, or a reduced risk of orthodontically induced root resorption.

From a strictly biomechanical perspective, the gingivally positioned LUMACA configuration showed a more uniform PDL deformation pattern and an initial displacement pattern closer to bodily translation than the other configurations under the simulated conditions. These characteristics should be interpreted as comparative mechanical findings rather than as evidence of clinical advantage. The present static FEA does not reproduce biological remodelling, time-dependent orthodontic tooth movement, patient-specific anatomical variability, tissue adaptation, or treatment-related adverse effects. Accordingly, no inference can be made regarding treatment efficiency, clinical predictability, periodontal safety, or prevention of orthodontically induced root resorption. These potential clinical implications require dedicated experimental and prospective clinical validation. In addition, a more apical slot position may increase interaction with adjacent soft tissues, with the potential for mucosal irritation or discomfort; this aspect also requires clinical evaluation.

### Limitations of the Study

As with all finite element studies, the results depend on assumptions regarding geometry, material properties, and boundary conditions. The PDL was modelled as an orthotropic elastic material with fixed material constants, and the only nonlinearity included was geometric. The load-magnitude analysis showed that this nonlinear contribution was negligible within the investigated range, such that the model behaved effectively linearly. The real PDL, however, exhibits a markedly nonlinear stress–strain response, viscoelasticity, and load-history dependence. None of these features was represented in the present model. Static loading was assumed, and time-dependent behaviour and biological remodelling were not considered. These constitutive simplifications limit the interpretation of absolute displacement and stress magnitudes, which should not be considered directly comparable with in vivo tooth mobility. Nevertheless, the three bracket configurations were compared under identical constitutive assumptions. The kinematic descriptors used for comparison are primarily governed by the relative translational and rotational stiffness of the ligament rather than by its absolute stiffness. Spatially non-uniform material behaviour, such as region-dependent stiffening generated by a nonlinear constitutive law, could alter the centre-of-rotation position and therefore warrants further investigation. Future studies should incorporate poroelastic, viscoelastic, and dissipative PDL formulations, dynamic loading, and experimental validation to assess the robustness of the present findings.

A further limitation is the two-dimensional plane-strain idealisation. The tooth–PDL–bone complex and the bracket–archwire system are inherently three-dimensional, and their mechanical behaviour depends on spatial interactions that cannot be fully reproduced in a planar model. In particular, the present formulation cannot capture clinically relevant three-dimensional phenomena such as torque expression and root torque control, mesiodistal tooth rotations, transverse tooth displacements, transverse archwire deflections, or the corresponding bracket–slot interactions. Similarly, asymmetries related to three-dimensional root morphology and the spatial organisation of the periodontal ligament cannot be represented. Moreover, the plane-strain assumption constrains out-of-plane deformation and may therefore increase the apparent stiffness of the simulated system relative to a fully three-dimensional model. Consequently, the absolute magnitudes of displacement, stress, and strain should be interpreted cautiously and primarily as comparative descriptors rather than as direct quantitative predictions of clinical tooth movement. This limitation is particularly relevant because bodily translation, torque expression, and rotational control are inherently three-dimensional mechanical phenomena. The present findings should therefore be regarded as an internally controlled comparison of bracket configurations under identical modelling assumptions. Future studies should employ fully three-dimensional finite element models incorporating realistic root morphology, explicit bracket–archwire contact, slot play, torque expression, and spatially oriented PDL properties to determine whether the relative biomechanical trends observed here are maintained under more clinically representative conditions.

A related limitation concerns model specificity. A single tooth geometry and a single set of material properties were considered, and no sensitivity analysis was performed for root morphology or PDL thickness. Both factors are known to influence biomechanical response, and PDL thickness in particular varies substantially in vivo. Load magnitude, however, was examined directly by repeating the simulations at five force levels between 0.25 and 2.00 N; within this range, movement pattern and configuration ranking remained unchanged. The absolute values reported should therefore be considered specific to the geometry and material properties of the present model. The comparison among configurations remains internally controlled because geometry, mesh, material properties, boundary conditions, and applied load were identical, with slot position as the principal variable. Nevertheless, the magnitude of the observed differences may vary with anatomy. Future parametric analyses should therefore examine root morphology and PDL thickness in conjunction with the planned three-dimensional extension of the model.

A further limitation concerns representation of the force system delivered by the archwire. The model applied a single force at the bracket slot, corresponding to an engaged wire transmitting no couple. In clinical use, however, a rectangular wire engaged with torque in a self-ligating bracket may transmit both force and couple. Because the model response was effectively linear over the load range examined, the response to a more complex force system could, in principle, be represented by superposition of the force-only solution and a pure-couple solution. A pure couple is independent of the point of application, and the tooth behaves approximately as a rigid body relative to the PDL; therefore, an identical couple would contribute similarly across the three configurations. Nevertheless, the absolute kinematic descriptors would change if torque expression were included. The present comparison should therefore be interpreted as isolating the effect of the force line of action and slot position. Explicit simulation of wire-slot interaction, including slot play and torque expression, is required in future studies.

An additional limitation concerns the structural behaviour of the connecting arm between the bracket base and slot. The present finite element model was designed to evaluate the biomechanical effects of slot position and did not specifically assess the mechanical resistance, deformation, or stress concentration within this component. Its structural integrity under higher orthodontic loads therefore requires dedicated mechanical testing.

Finally, the study is exclusively computational. No in vitro mechanical testing was performed to validate the predicted displacement patterns, force transmission, or structural response of the bracket–tooth system, and no clinical data are available to determine whether the differences observed in the finite element model translate into differences in actual orthodontic tooth movement. The present results should therefore not be interpreted as evidence of improved clinical effectiveness, treatment predictability, biological safety, or reduced risk of root resorption. Experimental validation and prospective clinical studies are required before these biomechanical findings can be translated into clinical recommendations.

## 6. Conclusions

Within the limitations of this two-dimensional static finite element model, bracket slot position substantially influenced the initial biomechanical response of the tooth–PDL complex. The gingivally bonded LUMACA configuration produced a centre of rotation located farther beyond the root apex, a more uniform PDL deformation pattern, and a smaller crown–apex differential displacement than the conventional configuration, indicating a mechanical response closer to bodily translation under the simulated loading conditions. These numerical findings indicate a more favourable biomechanical response associated with the modified force-application geometry under the simulated conditions; however, their clinical significance remains to be established.

Further in vitro mechanical testing is required to validate force transmission, bracket–archwire behaviour, structural response, and the predicted displacement patterns. Subsequently, controlled in vivo clinical trials will be necessary to determine whether these numerical biomechanical differences translate into clinically meaningful improvements in tooth-movement control, treatment predictability, and overall orthodontic performance.

## Figures and Tables

**Table 1:** Isotropic material parameters.

	Young’s Modulus (GPa)	Poisson Ratio
Tooth	18.3	0.32
Cortical bone	17.3	0.28
Trabecular bone	0.64	0.28
Titanium	105	0.33

**Table 2:** Orthotropic elastic constants adopted for the PDL. The first material axis of orthotropy is oriented transversely relative to the periodontal ligament, the secondary axis aligns longitudinally, and the tertiary axis is established to complete the orthogonal material reference frame. The constants are fixed and single valued: no material nonlinearity is introduced.

PDL Material Parameters	Value
Young’s modulus	
E_1_	0.48 MPa
E_2_	0.2 MPa
E_3_	0.2 MPa
Poisson ratio	
ν_12_	0.45
ν_23_	0.42
ν_13_	0.42
Shear modulus	
G_12_	0.165 MPa
G_23_	0.07 MPa
G_13_	0.07 MPa

**Table 3:** Displacement of the coronal and apical probe points for the three bracket configurations at five force levels. The values are displacement magnitudes, in which the proportionality to the applied load was verified; the movement descriptors quoted in the text are computed from the components along the direction of the load at the reference value of 1.47 N and, by the linearity demonstrated here, are independent of the load magnitude.

Configuration	F (N)	|*u_c_*| (µm)	|*u_a_*| (µm)	Δ = |*u_c_*| − |*u_a_*| (µm)	|*u_a_*|/|*u_c_*|
LUMACA, standard	0.25	0.07517	0.03653	0.03864	0.4860
	0.5	0.15034	0.07306	0.07728	0.4860
	1	0.30068	0.14612	0.15456	0.4860
	1.47	0.44200	0.21480	0.22720	0.4860
	2	0.60136	0.29224	0.30912	0.4860
LUMACA, gingival	0.25	0.04109	0.03244	0.00864	0.7896
	0.5	0.08217	0.06488	0.01729	0.7896
	1	0.16435	0.12977	0.03458	0.7896
	1.47	0.24159	0.19076	0.05083	0.7896
	2	0.32870	0.25954	0.06916	0.7896
Conventional	0.25	0.12598	0.03106	0.09492	0.2465
	0.5	0.25197	0.06212	0.18985	0.2465
	1	0.50394	0.12424	0.37970	0.2465
	1.47	0.74080	0.18264	0.55816	0.2465
	2	1.00790	0.24849	0.75941	0.2465

**Table 4:** Mesh convergence study; the LUMACA bracket in the standard position under 1.47 N. The integrated traction on the tooth–ligament interface has the exact value 1.47 N, so its departure from that value measures the discretisation error directly.

Mesh	Max Element Size (mm)	Elements Across the PDL	DoF	*u_c_* (µm)	*r* = *u_a_*/*u_c_*	∫traction (N)	Error vs. 1.47 N (%)
M1	0.1	~3	314,512	0.44201	0.48596	1.3734	6.57
M2	0.0667	~6	699,464	0.44204	0.48595	1.4353	2.36
M3	0.05	~8	1,236,658	0.44205	0.48596	1.4472	1.55
M4	0.033	~12	2,770,942	0.44206	0.485952	1.4577	0.837

## Data Availability

All data supporting the findings of this study are available within the paper.
